# Timed Deletion of *Twist1* in the Limb Bud Reveals Age-Specific Impacts on Autopod and Zeugopod Patterning

**DOI:** 10.1371/journal.pone.0098945

**Published:** 2014-06-03

**Authors:** David A. F. Loebel, Angelyn C. C. Hor, Heidi K. Bildsoe, Patrick P. L. Tam

**Affiliations:** 1 Embryology Unit, Children's Medical Research Institute, Westmead, New South Wales, Australia; 2 Sydney Medical School, The University of Sydney, Sydney, New South Wales, Australia; Instituto Gulbenkian de Ciência, Portugal

## Abstract

*Twist1* encodes a transcription factor that plays a vital role in limb development. We have used a tamoxifen-inducible Cre transgene, *Ubc*-CreERT2, to generate time-specific deletions of *Twist1* by inducing Cre activity in mouse embryos at different ages from embryonic (E) day 9.5 onwards. A novel forelimb phenotype of supernumerary pre-axial digits and enlargement or partial duplication of the distal radius was observed when Cre activity was induced at E9.5. Gene expression analysis revealed significant upregulation of *Hoxd10*, *Hoxd11* and *Grem1* in the anterior half of the forelimb bud at E11.5. There is also localized upregulation of *Ptch1*, *Hand2* and *Hoxd13* at the site of ectopic digit formation, indicating a posterior molecular identity for the supernumerary digits. The specific skeletal phenotypes, which include duplication of digits and distal zeugopods but no overt posteriorization, differ from those of other *Twist1* conditional knockout mutants. This outcome may be attributed to the deferment of *Twist1* ablation to a later time frame of limb morphogenesis, which leads to the ectopic activation of posterior genes in the anterior tissues after the establishment of anterior-posterior anatomical identities in the forelimb bud.

## Introduction

The vertebrate limb is patterned by the interplay of the regionalized activities of secreted morphogens and transcription factors that drive tissue morphogenesis. Together they provide the positional information defining tissue patterns in the proximal-distal (PD), dorsal-ventral (DV), and anterior-posterior (AP) axes. AP patterning is influenced by the activity of the zone of polarizing activity (ZPA), located in the posterior-proximal limb bud. The ZPA expresses the signaling ligand sonic hedgehog (SHH) and imparts posterior characteristics to the limb tissues, controlling digit number and identity. Transplantation of the ZPA or ectopic presentation of SHH to the anterior limb bud results in the formation of duplicate post-axial limb elements (e.g. posterior digits) on the pre-axial side [1]. Loss of *Shh* function results in the loss of the ulna and most of the digits due to the absence of posteriorizing morphogenetic signals [2]. Maintenance of proper SHH signaling requires interactions with the BMP and FGF signaling pathways. Regulation of SHH signaling in the limb bud requires the activity of the BMP antagonist *Grem1*, and FGF signaling from the apical ectodermal ridge (AER). BMP signaling restricts the expression of *Shh* to the posterior domain by antagonizing FGF signaling. The antagonist, *Grem1*, is itself regulated via feedback inhibition from FGF signaling [3], [4], [5], [6]. Within the signaling milieu, a network of transcription factors regulates the expression of pathway components and mediates the response to these signals. *Hoxa* and *Hoxd* genes are important for regulating the number and arrangement of digits, development of the radius and ulna [9], and for initiating the SHH-BMP-FGF signaling loop [10]. Deletion of *Hoxd1-10* leads to ectopic *Hoxd11-13* expression in the mesenchyme in the anterior part of the limb bud and consequently the loss of AP asymmetry [11]. *Hoxa/d* genes may therefore act combinatorially in the AP patterning of the limb.

The *Twist1* gene, which encodes a basic helix-loop-helix (bHLH) transcription factor, is broadly expressed in the limb mesenchyme from early outgrowth of the limb bud. By E12.5, *Twist1* expression is restricted to the proximal limb tissues, interdigital mesenchyme and interphalangeal joints [12]. Loss of *Twist1* function curtails forelimb bud outgrowth, which is accompanied by reduced FGF, SHH and BMP signaling, but has less severe effects on the hindlimb buds [12]. Conditional loss of *Twist1* function mediated by *Prrx1*-Cre after the initiation of limb outgrowth [13], [14] results in disrupted AP patterning of the limb leading to the development of supernumerary digits, loss of the radius and reductions in humerus, scapula and clavicle development [13], [14]. Hand2 binds to and antagonizes the actions of Twist1, also triggering the formation of additional digits when it is ectopically expressed [7], [8]. Ablation of *Twist1* activity only in the anterior mesenchyme of the forelimb bud leads to posteriorization of anterior skeletal elements, including mirror-image digit duplications and the acquisition of an ulnar-like morphology by the radius [15]. This phenotype is associated with the down-regulation of *Alx* gene family members and *Gli3* in the anterior tissue of the forelimb bud, and a concomitant expansion of *Hoxd13* and *Grem1* expression domains into the anterior region of the limb bud.

Here, we have examined the impact of loss of *Twist1* on the patterning of limb tissues after the limb bud has initiated outgrowth. Following the induction of Cre activity at E9.5, the limb displayed a partial radius duplication and preaxial polydactyly, but without morphological posterior transformation. The dysmorphology of the autopod and zeugopod is accompanied by upregulation of SHH signaling, *Grem1* and posterior transcription factors in the anterior limb bud, which is detectable at E11.5 but not E10.5.

## Materials and Methods

### Mouse strains and tamoxifen injection

Twist1^del/+^; *Ubc*-Cre-ERT2 mice were generated by crossing *Twist1*-del [16] and *Ubc*-CreERT2 strains [17]. *Ubc*-CreERT2 mice were obtained from the Jackson Laboratory. Mice and embryo yolk sacs were genotyped for *Twist1* and *Cre* as previously described [16]. To produce conditional knockout embryos, *Twist1^del/+^*; *Ubc*-Cre-ERT2 mice were crossed with *Twist1^3loxPneo/3loxPneo^* mice [18]. Pregnant females with injected intraperitoneally with 4 mg (E9.5 injection) or 8 mg (E10.5 or later injection) tamoxifen at embryonic day (E) 9.5–14.5. This study was carried out in accordance with the Australian Code for the Care and Use of Animals for Scientific Research and was approved by the Children's Medical Research Institute/Children's Hospital Westmead Animal Ethics Committee (project number C230). All efforts were made to minimize animal suffering.

### Immunofluorescence

Staining for Twist1 protein was carried out on frozen sections of E11.5 forelimb buds using mouse monoclonal anti-Twist1 antibody 2C1A (Abcam ab50887, 1/50 dilution) and AlexaFluor 488 conjugated donkey anti-mouse secondary antibody (Life Technologies) as described [15]. Stained sections were imaged on a Zeiss Axio Imager M1. For bone and cartilage staining, embryos were collected in water at E17.5, skinned and stained as whole specimens as described previously [15], [19]. Specimens were stored in 80% glycerol and limbs were photographed with a Leica microscope and SPOT digital camera.

### RNA extraction and RT-PCR

Embryos were collected at E11.5, forelimb buds dissected into anterior and posterior halves using 30G needles and frozen in pairs in liquid nitrogen. Total RNA was extracted with the RNeasy Micro kit (QIAGEN) for analysis of coding genes or RNeasy miRNA Micro kit (QIAGEN) for miRNA analysis. Reverse transcription with oligo-dT priming was performed with SuperScript III (Life Technologies) and quantitative (q)RT-PCR carried out using Sybr green and Platinum Taq polymerase (Life Technologies). miRNA analysis was performed using N-Code (Life Technologies) for poly-A tailing, reverse transcription and amplification. qRT-PCR was performed on a Corbett RotorGene thermocycler. The following qRT-PCR primers were used:

*Hand2-F* 5′-acatcgcctacctcatggatctgct-3′

*Hand2-R* 5′- ctgtccggcctttggttttcttgt-3′

*Hoxd10*-F 5′-aagacaggagctgcctggctga-3′

*Hoxd10*-R 5′-aacgctcttactgatctctaggcggc-3′

*Hoxd11*-F 5′-tggttccagaatcgcaggatgaaa-3′

*Hoxd11*-R 5′-cccgagttgaaggcgagaagaaat-3′

*Hoxd13*-F 5′- taccagtcctggacgctagccaa-3′

*Hoxd13*-R 5′-taaggcacccttttcttccttccc-3′

*Grem1*-F 5′-tagcggcgctctccttcgtctt-3′

*Grem1*-R 5′-tttttcccctcagctgttggcagta-3′

*Ptch1*-F 5′-gggttctcaatggactggttctgct-3′

*Ptch1*-R 5′-caaaccggacgacacttggagg-3′

*Twist1*-F 5′- ccctcggacaagctgagcaaga -3′

*Twist1*-R 5′- atcctccagacggagaaggcgta-3′

*Polr2a*-F 5′-gcaccacgtccaatgatat-3′

*Polr2a*-R 5′-gtgcggctgcttccataa-3′

*miR10b*-F 5′-taccctgtagaaccgaatttgtg-3′

*miR191*-F 5′-caacggaatcccaaaagcagctg-3′

### Whole mount in situ hybridization

Embryos were collected at E10.5 and E11.5 and fixed in 4% paraformaldehyde. In situ hybridization was carried out either manually or using a Intavis InSituPro robotic system according to methods previously described [16], [20]. Digoxygenin labeled riboprobes were generated using Ampliscribe (Epicentre) from cDNA clones containing fragments of the *Hand2*, *Hoxd13* and *Ptch1* transcripts.

## Results

### Generation of embryos with Twist1 deleted at specific developmental ages

We utilized mice carrying a broadly expressed tamoxifen-activated Cre transgene, *Ubc*-CreERT2 [17] to generate timed deletions of *Twist1* at specific stages of embryonic development. To reveal the age/stage-specific impact of loss of *Twist1* activity on development, pregnant mice were injected with tamoxifen or oil at E9.5–E14.5 and embryos were collected at E17.5. Embryos from mothers injected with oil only and wild type embryos from tamoxifen injected mothers developed normally (Fig. S1 A, B). After injection at E9.5 (TAM E9.5) 4/7 CKO embryos were small and underdeveloped (Fig. S1 C). Consistent with previous studies, all TAM E9.5 embryos (7/7) displayed craniofacial defects including a cleft face and misshapen cranial region (Fig. S1 C, D) and TAM E10.5 embryos displayed a misshapen cranium and shortened snout (7/7 embryos, Fig. S1 E), whereas TAM E12.5 embryos showed no obvious head and face abnormalities (Fig. S1 F). Limbs of TAM E9.5 embryos appeared shorter than wild-type embryos and preaxial supernumerary digits were prominent in all embryos (Fig. S1 C′, D′). Polydactyly was not observed in embryos from mothers injected at later stages, but embryos from E10.5–E12.5 injected mothers (TAM E10.5, 7/7 embryos; TAM E11.5 3/4 embryos; TAM E12.5, 5/5 embryos) showed a curvature of the digits (Fig. S1 E, F; Table 1). The forelimb phenotypes observed in TAM E9.5 embryos differed from those previously observed in *Twist1* conditional mutant limbs, prompting us to examine this unique phenotype further.

**Table 1:** Comparison of *Twist1*:*Ubc*-CreERT2 CKO forelimb skeletal phenotypes, from mothers injected at E9.5–E12.5 with *Twist1*: *Mesp1*-Cre and *Prrx1*-Cre CKO embryos and s*ka10* (*charlie chaplin*) mutant embryos.

	TAM E9.5	TAM E10.5	TAM E11.5	TAM E12.5	*Mesp1*-Cre	*Prrx1*-Cre	*ska10*/*ska10*	*ska10*/-
Number of limbs analysed	4	10	4	4	N/A	N/A	N/A	N/A
Autopod								
Supernumerary digits/fragments	Y				Y	Y		
Mirror transformation					Y	Y	Y	
Bifid digits					Y	Y	Y	
Fewer digits							Y	Y
Extra carpals	Y				Y	Y		
Curved digits	Y	Y	Y			Y	Y	
Reduced/delayed ossification	Y	Y	Y	Y	Y	Y		Y
Zeugopod and Stylopod								
radius: partial duplication or thickening	Y							
Radius: ulnarization					Y	Y		Y
Radius: loss						Y	Y	
Ulna: malformed	Y							
Humerus: tuberosity reduced or lost	Y	Y			Y		Y	
Humerus: short/malformed					fused to scapula	Y		Y
Scapula/clavicle: reduced/malformed	Y	variable			small spine	Y	Y	Y

Y, presence of phenotype; N/A: published work, not applicable,

References: *Mesp1*-Cre data [15]; *Prrx1*-Cre, *ska10* data [13], [14].

**Figure 1:**
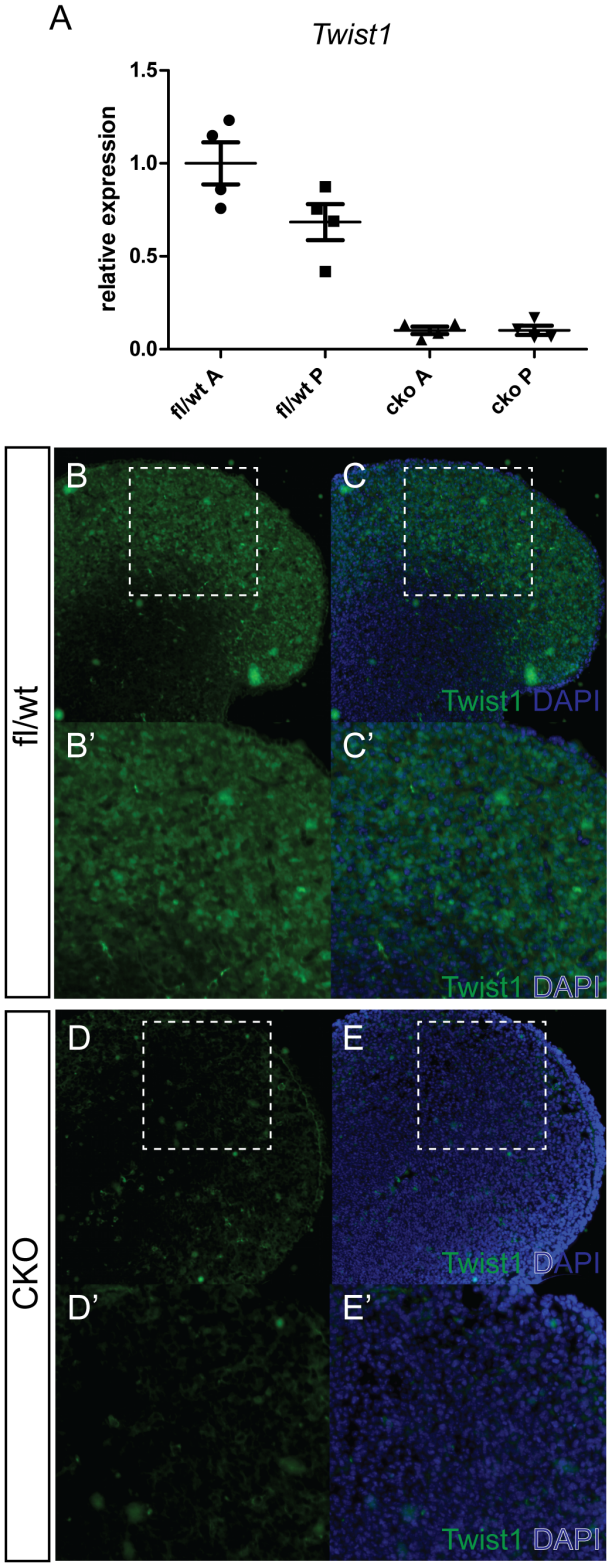
Efficient ablation of *Twist1* expression in conditional knockout embryos. (A) qRT-PCR analysis of RNA from anterior (A) and posterior (P) halves of forelimb buds collected E11.5 from mothers injected with tamoxifen at E9.5. n = 4 pairs of forelimb buds per genotype. (B–E, B′–E′.) Immunostaining with anti-Twist1 monoclonal antibody (green) of cryosectioned wild type (fl/wt) (B, C, B′, C′) and conditional knockout (CKO) (D, E, D′, E′) E11.5 forelimb buds of embryos harvested from mothers injected with tamoxifen at E10.5. (C, C′, E, E′) Merged images showing counterstaining with DAPI (blue). (B′–E′) Higher magnification images of the boxed regions in B–E.

We used qRT-PCR and immunofluorescent staining to determine the efficiency of depletion of *Twist1* in limb buds. Quantitative RT-PCR analysis of RNA from dissected forelimb buds collected at E11.5, following tamoxifen injection at E9.5 revealed very low levels of Twist1 transcripts in conditional knockout (CKO) embryos (Fig. 1 A). Immunostaining of E11.5 limb buds for Twist1 protein revealed that, whereas Twist1 was detected widely in the limb bud mesenchyme of control embryos (Fig. 1 B, C, B′, C′), specific nuclear staining was almost undetectable in CKO limb bud tissues 24 hours after tamoxifen injection at E10.5 (Fig. 1 D, E, D′, E′). These results confirm that *Ubc*-CreERT2 activity can efficiently ablate *Twist1* following tamoxifen treatment.

### Conditional Twist1 deletion at E9.5–E12.5 causes different limb defects

#### The forelimb shoulder and stylopod phenotype

**Figure 2:**
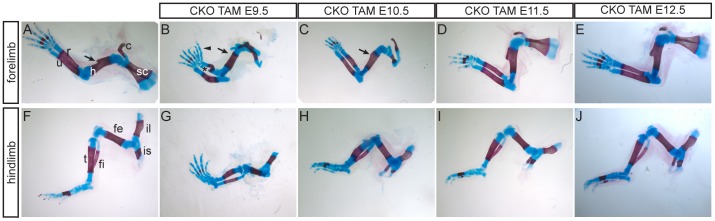
Limb skeletal phenotypes of timed conditional knockout embryos at E17.5. (A–E) forelimbs, (F–J) hindlimbs. (A, F) Wild-type, (B–E, G–J) conditional knockout embryos harvested from mothers injected with tamoxifen at E9.5 (B, G), E10.5 (C, H), E11.5 (D, I) or E12.5 (E, J). Asterisk in B indicates additional cartilage attached to radius; arrowhead indicates supernumerary digits, arrows in (A–C) marks the deltoid tuberosity, which is absent in (B). Abbreviations: c, clavicle; fe, femur; fi, fibula; h, humerus; il, ilium; is, ischium; r, radius; t, tibia; u, ulna.

In TAM E9.5 embryos, the clavicle was absent and the scapula was malformed, consisting of two separate fragments (Fig. 2 B, compare with wild-type Fig. 2 A; 4/4 limbs). The scapula phenotype varied in TAM E10.5 embryos. In some limbs the scapula resembled that of TAM E9.5 embryos (Fig. 2 C, 4/10 limbs), but in others a more complete scapula was formed, but with reduced ossification (6/10 limbs, Table 1). The scapula and clavicle of TAM E11.5 and TAM E12.5 embryos were similar to wild-type embryos (Fig. 2 D, E). The scapula and clavicle abnormalities of TAM E9.5 and TAM E10.5 embryos was more dramatic than that seen when *Twist1* was deleted specifically from the anterior limb bud by *Mesp1*-Cre (Table 1) [15]. The humerus of TAM E9.5 embryos lacked the deltoid tuberosity, which was also reduced in TAM E10.5 embryos (Fig. 2 A–C, 4/4 limbs). Loss of the deltoid tuberosity was also seen in *Mesp1*-Cre CKO embryos and *ska10* point mutant embryos, but a more severe reduction in the humerus occurred in *Prrx1*-Cre CKO embryos (Table 1) [13]–[15].

#### The forelimb zeugopod phenotype

**Figure 3:**
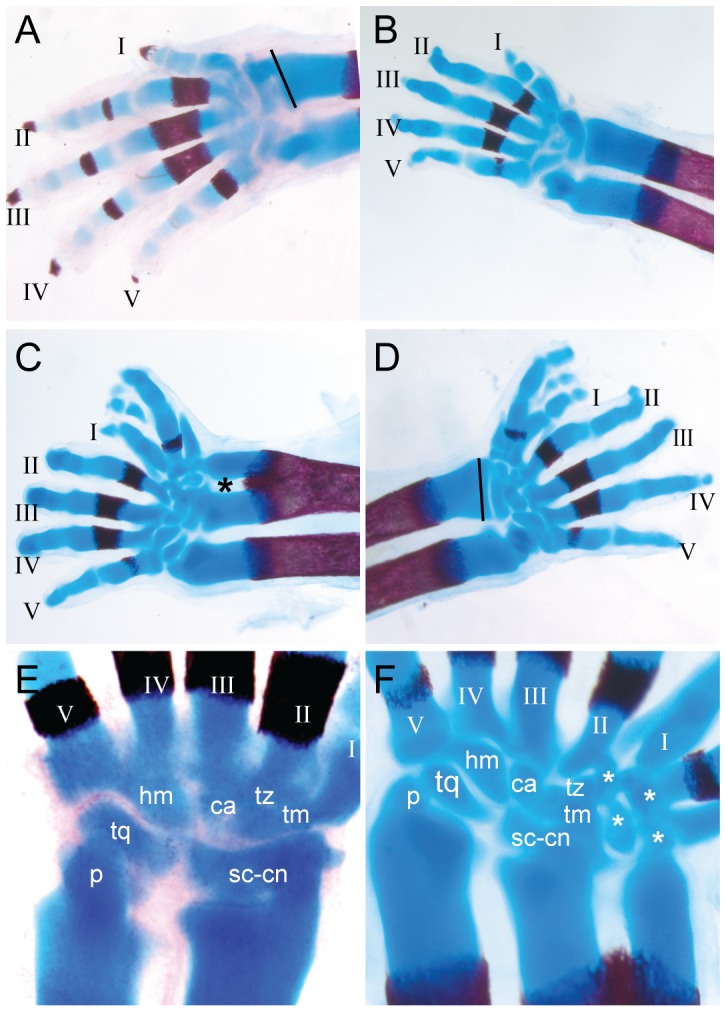
Autopod and distal zeugopod phenotypes in TAM E9.5 embryos. (A, E) Wild-type, (B–D, F) CKO. Embryos were harvested at E17.5 from mothers injected with tamoxifen at E10.5 (B) or E9.5 (C, D, F) and stained with alcian blue or alizarin red. Digit identities are indicated with Roman numerals. Black asterisk indicates partial duplication of the distal radius, white asterisk indicates additional carpal elements. Black line shows the width of the distal radius in the TAM E9.5 embryo (D), superimposed on wild-type (A). Abbreviations: ca, capitate; hm, hamate; p, pisiform; r, radius; s, scapula; sc-cn, scaphoid-centrale; tq, triquetral; tm, trapezium; tz, trapezoid; u, ulna.

The radius and ulna of TAM E9.5 embryos were bent and shorter than in normal embryos. Extra cartilage was formed at the distal end of the radius resulting in either a partial duplication (3/4 limbs), or broadening of the distal end of the radius (1/4 limbs; Fig. 2 B, Fig.3 C, D, compared with Fig. 3A). This was not previously observed in *Mesp1*-Cre or *Prrx1*-Cre CKO embryos (Table 1; [13]–[15]) and there was no indication of the “ulnarization” of the radius that was observed in *Mesp1*-Cre *Twist1* CKO embryos [15]. No extra radius cartilage was found in TAM E10.5–TAM E12.5 embryos (Fig. 2 C–E).

#### The forelimb autopod phenotype

Ossification of carpals of TAM E9.5 and TAM E10.5 embryos was delayed or reduced compared to control embryos (Fig. 3 A–D), a common feature of other *Twist1* CKO limbs (Table 1). Digits displayed a curved morphology and the joints between phalanges were not well defined. One or two complete supernumerary digits with no clear AP identity were present on the pre-axial side in TAM E9.5 limbs (4/4 limbs), and additional metacarpal fragments were seen in 2/4 limbs (Fig. 2 B, Fig. 3C, D). Supernumerary digits were not found in TAM E10.5 embryos (0/10 limbs; Fig. 3 B). One to four extra carpal elements, also of uncertain identity, were associated with the additional digits (Fig. 3 E, F). These forelimb autopod abnormalities differed from the evident posterior transformation observed in *Mesp1*-Cre and *Prrx1*-Cre Twist1 CKO limbs and the loss of digits seen in other mutant embryos (Table 1; [13]–[15]).

#### Hindlimb phenotype

In the hindlimb of TAM E9.5 embryos, the ischium was absent and the ilium was reduced (Fig. 2 F, G; 4/4 limbs). Ossification was retarded in the tibia, fibula and femur in TAM E9.5 and TAM E10.5 embryos (Fig 2 F, H). Digits were curved in TAM E10.5–12.5 hindlimbs (Fig. 2 H–J, compare to Fig. 2 F). Hindlimb polydactyly with incomplete penetrance is observed in *Twist1^+/−^* mice [12], [21] and therefore is not unique to the *Ubc*-CreERT2 CKO mutants. No exacerbation of this phenotype was observed in the hindlimbs of these CKO embryos.

### Twist1 restricts posterior gene expression in forelimb buds

**Figure 4:**
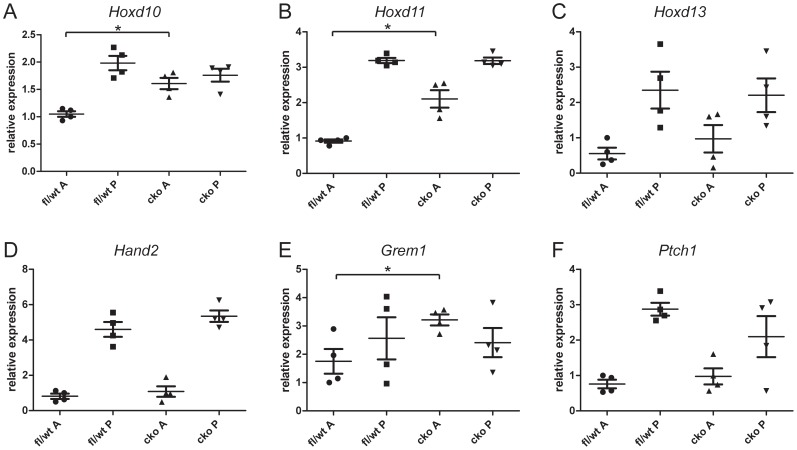
Quantitative RT-PCR analysis of gene expression in forelimb buds. Pairs of forelimb buds dissected from control (fl/wt) or conditional knockout (cko) E11.5 embryos harvested from mothers injected with tamoxifen at E9.5 were dissected into anterior (A) and posterior (P) halves and qRT-PCR was performed for *Hoxd10* (A), *Hoxd11* (B), *Hoxd13* (C), *Hand2* (D), *Grem1* (E) and *Ptch1* (F). Asterisk indicates p<0.05 in two-tailed t-test between anterior halves of fl/wt and cko limb buds. Reference gene was *Polr2a*. n = 4 embryos for each tissue and genotype.

To further investigate the forelimb autopod and zeugopod defects in TAM E9.5 embryos, we examined the expression of genes that are normally differentially expressed in anterior and posterior parts of the forelimb bud by qRT-PCR (n = 4 embryos, collected at E11.5, for each genotype and transcript measured). In control embryos, *Twist1* is preferentially expressed in the anterior half of the forelimb bud, and in TAM E9.5 embryos its expression was reduced to very low levels in both anterior and posterior fragments (Fig. S1 A). *Hoxd10* and *Hoxd11* are normally expressed most strongly in the posterior part of forelimb bud. In forelimb buds from TAM E9.5 CKO embryos, both genes remained strongly expressed in the posterior half of the bud, but were significantly up-regulated in the anterior half of the limb bud (Fig 4 A, B). This is consistent with a previous observation that *Hoxd11* is upregulated in compound *Twist1^ska10^* ^/-^ mutant limb buds [13]. In contrast, *Hoxd13* was not significantly upregulated in CKO anterior limb bud tissues compared to controls (Fig 4 C). *Hand2*, encoding a bHLH transcription factor normally expressed in the posterior limb bud also showed no significant upregulation (Fig. 4 D). *Grem1*, encoding a BMP antagonist that depends on *Hoxd* activity to initiate its expression [10] showed significantly higher expression in CKO anterior forelimb bud tissue than in control forelimb buds (Fig. 4 E). The expression of *Ptch1*, a target of SHH signaling, was not increased in CKO anterior forelimb relative to controls (Fig. 4 F) but was reduced in the posterior tissue of some CKO specimens. This suggested that there was a globally reduced level of SHH signaling in some TAM E9.5 CKO limb buds, which was also encountered in *Twist1*-null embryos [12].

*miR10b* is a transcriptional target of Twist1 in cancer cells and is involved in repression of *Hoxd10* expression [22], [23], suggesting that Twist1 may regulate *Hoxd10* expression via control of this miRNA. qRT-PCR analysis showed that *miR10b* is expressed in E11.5 forelimb bud tissues (Fig. S2) but its expression did not change significantly in either the anterior or posterior fragments of the CKO limb buds. Therefore, lack of regulation by *miR10b* may not account for *Hoxd10* upregulation in TAM E9.5 limb buds.

**Figure 5:**
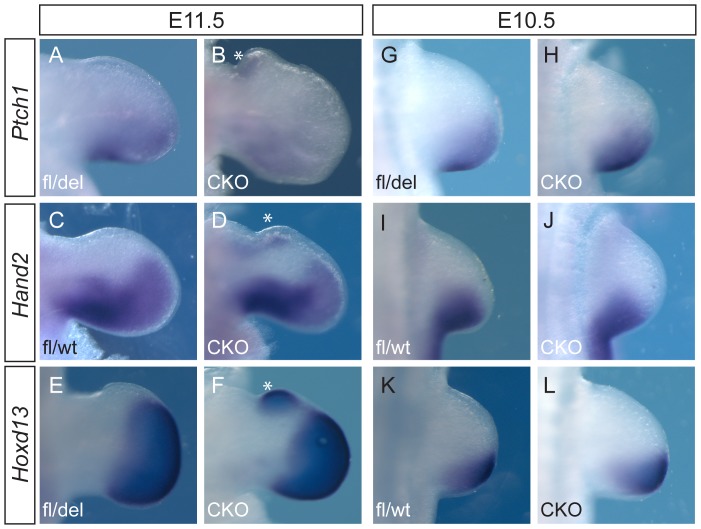
Whole mount in situ hybridization of the forelimb bud. Expression of *Ptch1* (A, B, G, H), *Hand2* (C, D, I, J) and *Hoxd13* (E, F, K, L) in wild-type (fl/wt) or heterozygous (fl/del) embryos (A, C, E, G, I, K) were compared to CKO embryos (B, D, F, H, J, L) collected at E11.5 (A–F) or E10.5 (G–L). Asterisk indicates ectopic anterior expression domains that may correspond to the site of formation of the extra digits.

Although we did not detect significant upregulation of *Hoxd13*, *Hand2* or *Ptch1* in anterior forelimb buds by qRT-PCR analysis, which indicated that there were no widespread differences in expression these genes between control and CKO forelimb buds, the range of expression values was greater in CKO anterior limb buds than in controls. This suggested that there may be subtle localized changes in gene expression. To test this possibility, we performed whole mount in situ hybridization of forelimb buds of TAM E9.5 embryos collected at E10.5 and E11.5. At E11.5, the *Ptch1* hybridization signal was weaker in CKO limb buds (3/4) than in wild type limb buds, consistent with our qRT-PCR results (Fig. 5 A, B). Localized ectopic expression of *Ptch1* (3/4 limb buds), *Hand2* (4/4 limb buds) and *Hoxd13* (4/4 limb buds) was found in the anterior tissue of the forelimb bud (Fig. 5 B, D, F, compared with Fig. 5 A, C, E). Ectopic expression was localized to a small anterior tissue swelling in CKO limb buds, possibly containing the precursors of the supernumerary digits. Ectopic expression of these genes was not seen at E10.5 (n = 2 limb buds for each; Fig. 5 G–L). These observations suggest that, although there was no apparent morphological posterior transformation, the ectopic digits had acquired a posterior molecular identity. However, despite the widespread loss of Twist1 protein by 24 hours after tamoxifen injection (Fig. 1), detectable ectopic expression of these posterior tissue markers did not occur until after E10.5.

## Discussion

### Stage-specific requirement for Twist1 in limb development

In this study we have used a tamoxifen-inducible Cre recombinase to ablate *Twist1* at different embryonic ages. The forelimb preaxial polydactyly and other skeletal defects observed in tamoxifen-treated embryos (Fig. 2, Fig. 3) differed from those previously reported in either *Mesp1*-Cre or *Prrx1*-Cre induced Twist1 CKOs (Table 1). Previous studies of *Twist1* and limb development have used tissue-specific Cre transgenes to delete *Twist1* in the newly formed anterior mesoderm, including the mesoderm that contributes to the head and anterior forelimb (*Mesp1*-Cre; [15], [24]) or in the limb buds shortly after initiation of outgrowth (*Prrx1*-Cre; [13], [14]). In this study, the ablation of *Twist1* activity between E9.5 and E10.5 results in the formation of extra digits on the preaxial side of the forelimb and a partial duplication or enlargement of the distal part of the radius, without any apparent posterior anatomical transformations of skeletal elements. The consequences of deleting *Twist1* after limb bud formation, which affects skeletal morphogenesis and differentiation but not AP patterning, are strikingly different from the mirror image duplications, bone loss and posterior transformations produced by the ablation of *Twist1* with *Mesp1*-Cre, which acts in the nascent upper trunk mesoderm, or with *Prrx1*-Cre, which acts in the early limb bud mesenchyme (Table 1; [13]–[15]). Data obtained from tamoxifen injection at E9.5 show that *Twist1* is no longer required for maintaining AP anatomical identity in the limb skeleton at this age. However, *Twist1* may still have a role in regulating digit number and the morphogenesis of the distal zeugopod, and for restriction of posterior gene expression.

### Twist1-dependent gene expression influences autopod and zeugopod patterning

The limb phenotypes seen in this study are reminiscent of those observed when Hand2 or Hand2-Twist1 dimers are mis-expressed during limb development, which differ from the consequences of Twist1 homodimer or Twist1-E2A heterodimer expression (Table 2). *Hand2* encodes a bHLH protein that can dimerize with Twist1 and, when over-expressed in the limb bud, causes the formation of extra digits and the mirror image duplication of digits [25] similar to those observed when *Twist1* was specifically deleted in the anterior compartment of the forelimb bud [15]. The formation of additional preaxial digits and malformations of the zeugopod do not require the ability of Hand2 to bind DNA [7], suggesting that Hand2 could exert these effects by interacting with another bHLH factor, such as Twist1. Consistent with this, enforced expression of tethered Twist1-Hand2 heterodimer results in the formation of supernumerary digits and additional radius structures (Table 2; [8]). Genetic interaction (in mice) and mis-expression studies (in chick) indicates that *Twist1* and *Hand2* antagonize each other's actions in the limb bud [8]. Since *Hand2* is normally expressed in the posterior forelimb bud, and *Twist1* primarily functions in regulating gene expression in the anterior forelimb bud, ectopic expression of *Hand2* could generate novel Twist1-Hand2 dimers that may compete with the endogenous Twist1-containing dimers for action, resulting in a loss of normal Twist1-dimer function. This contrasts with the effects of over-expression of Twist1 monomer or homodimer, which similarly cause reductions and malformations of the zeugopod and stylopod and with the less drastic effects of the over-expression of Twist1-E12 heterodimer (Table 2). It has previously been noted that expression of Twist1 homodimers promotes *Fgfr2* expression and differentiation in calvarial osteoblasts, whereas Twist1-E2A heterodimers repress it [26]. Therefore, changes in dimer contents could account for the similar phenotype of heterodimer over-expression and conditional knockout studies.

**Table 2:** Comparison of *Twist1*:*Ubc*-CreERT2 CKO embryo forelimb phenotype after tamoxifen injection at E9.5 with embryos that overexpress Twist1 monomer or Twist1-Hand2, Twist1-E12 or Twist1-Twist1 tethered dimers. [30].

	TAM E9.5	Twist1-Hand2	Twist1 monomer	Twist1-Twist1	Twist1-E12
Autopod					
Supernumerary digits/fragments	Y	Y			
Fewer digits				Y	
Extra carpals	Y				
Curved digits	Y				
Reduced/delayed ossification	Y				
Zeugopod and Stylopod					
radius: partial duplication or thickening	Y	Y			
Radius: loss/reduction			small	Y	
Ulna: malformed	Y		small	Y	small
Humerus: tuberosity reduced or lost	Y	Y	Y	Y	Y
Humerus: short/malformed			Y	Y	
Scapula/clavicle: reduced/malformed	Y		Y		

Loss of *Hoxa13* and *Hoxd13* function results in missing or truncated digits [9]. Ectopic anterior expression of *Hoxd11-13* together with deletion of *Hoxd1-10* [11] causes ectopic upregulation of *Shh* expression, resulting in posteriorization of the limb structures [11]. Hand2 is also required for *Shh* activity in the posterior tissues of the limb bud and, along with Hoxd13, binds to a distant upstream regulator of *Shh* [27]. It is therefore likely that the ectopic upregulation of *Hand2* and *Hoxd* genes along with the disruption to BMP-SHH feedback [4] due to ectopic *Grem1* expression, underlies the localized elevation of SHH activity (revealed by *Ptch1* expression) and consequently the formation of extra pre-axial digits in CKO embryos.

### Timing of Twist1 deletion influences the phenotypic response to downstream gene expression

In chick limbs, exposure of the anterior wing bud to SHH results in the formation of additional digits that mirror those on the posterior side. The induction of ectopic digits is sensitive to the time of exposure to SHH [28] with early, prolonged exposure leading to duplications of postaxial digits on the anterior side. In mouse embryos, individual digits show a differential sensitivity to timing of loss of *Shh*, with digit 2 only being lost when *Shh* is deleted early (E9.5 tamoxifen injection) and digit 3 affected when *Shh* deletion is initiated as late as E10.5 [29]. Together, these data suggest that the timing of onset and duration of exposure to SHH influences the formation of specific digits to different degrees and determines the morphological identity of the digits. In the present study, ectopic SHH signaling was detected at E11.5 but not E10.5. Ectopic Shh signaling commencing in this time window may cause the formation of additional preaxial digits and zeugopod elements but not posterior transformation. Therefore, although the molecular response to loss of *Twist1* activity is similar when *Twist1* is deleted at different stages [13]–[15], the timing of this response may determine the phenotypic impact on the skeletal elements.

## Supporting Information

Figure S1 **Timed deletion of** ***Twist1*** **by tamoxifen inducible Cre recombinase.** Embryos harvested at E17.5 from oil (A) or tamoxifen (B–F) injected mothers. (B) Twist1^flox/+^ wild-type (WT) control. (C, D) Conditional knockout (CKO) embryos from mothers injected with tamoxifen at E9.5. (C′, D′) high magnification views showing supernumerary digits in TAM E9.5 embryos (white arrowheads). (E, F) CKO embryos from mothers injected ar E10.5 and E12.5. (F) Asterisk in (D) indicates cleft face; black arrowheads in (E, F), curved digits. Panels A, B, C, D, E, and F were photographed at the same magnification.
(TIF)

Figure S2 ***miR10b*** **is not significantly downregulated in conditional mutant limb buds.** qRT-PCR for *miR10b* in anterior (A) and posterior (P) halves of E11.5 forelimb buds dissected from control (fl/wt) and TAM E9.5 (cko) embryos. Reference gene was *miR191*. N = 3 for each genotype.
(TIF)
