# Supplementary figures and images for: Timed Deletion of Twist1 in the Limb Bud Reveals Age-Specific Impacts on Autopod and Zeugopod Patterning
Source: PLoS One. 2014 Jun 3;9(6):e98945. doi: 10.1371/journal.pone.0098945 (PMC4044014; doi:10.1371/journal.pone.0098945)

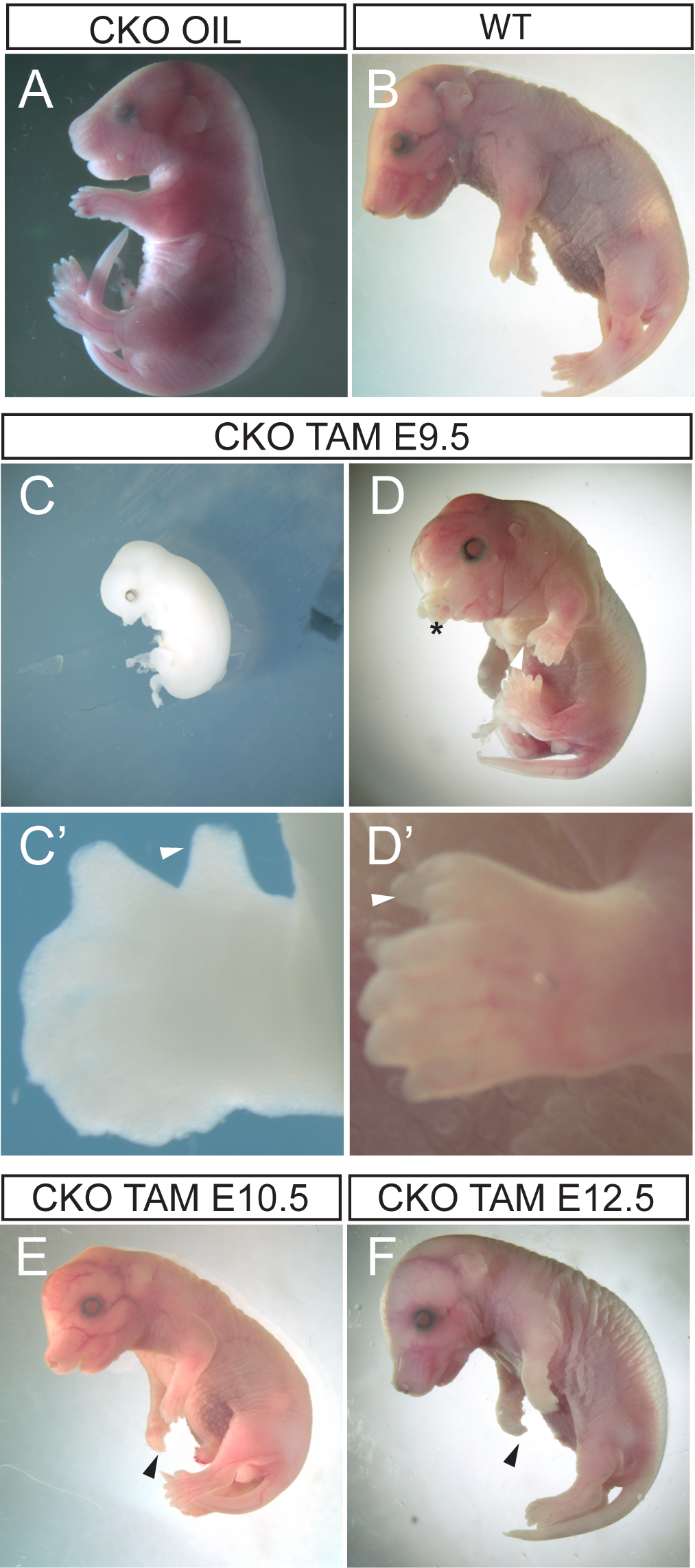

Supplement: Figure S1 — Timed deletion of Twist1 by tamoxifen inducible Cre recombinase. Embryos harvested at E17.5 from oil (A) or tamoxifen (B–F) injected mothers. (B) Twist1flox/+ wild-type (WT) control. (C, D) Conditional knockout (CKO) embryos from mothers injected with tamoxifen at E9.5. (C′, D′) high magnification views showing supernumerary digits in TAM E9.5 embryos (white arrowheads). (E, F) CKO embryos from mothers injected ar E10.5 and E12.5. (F) Asterisk in (D) indicates cleft face; black arrowheads in (E, F), curved digits. Panels A, B, C, D, E, and F were photographed at the same magnification. (TIF) [file pone.0098945.s001.tif]

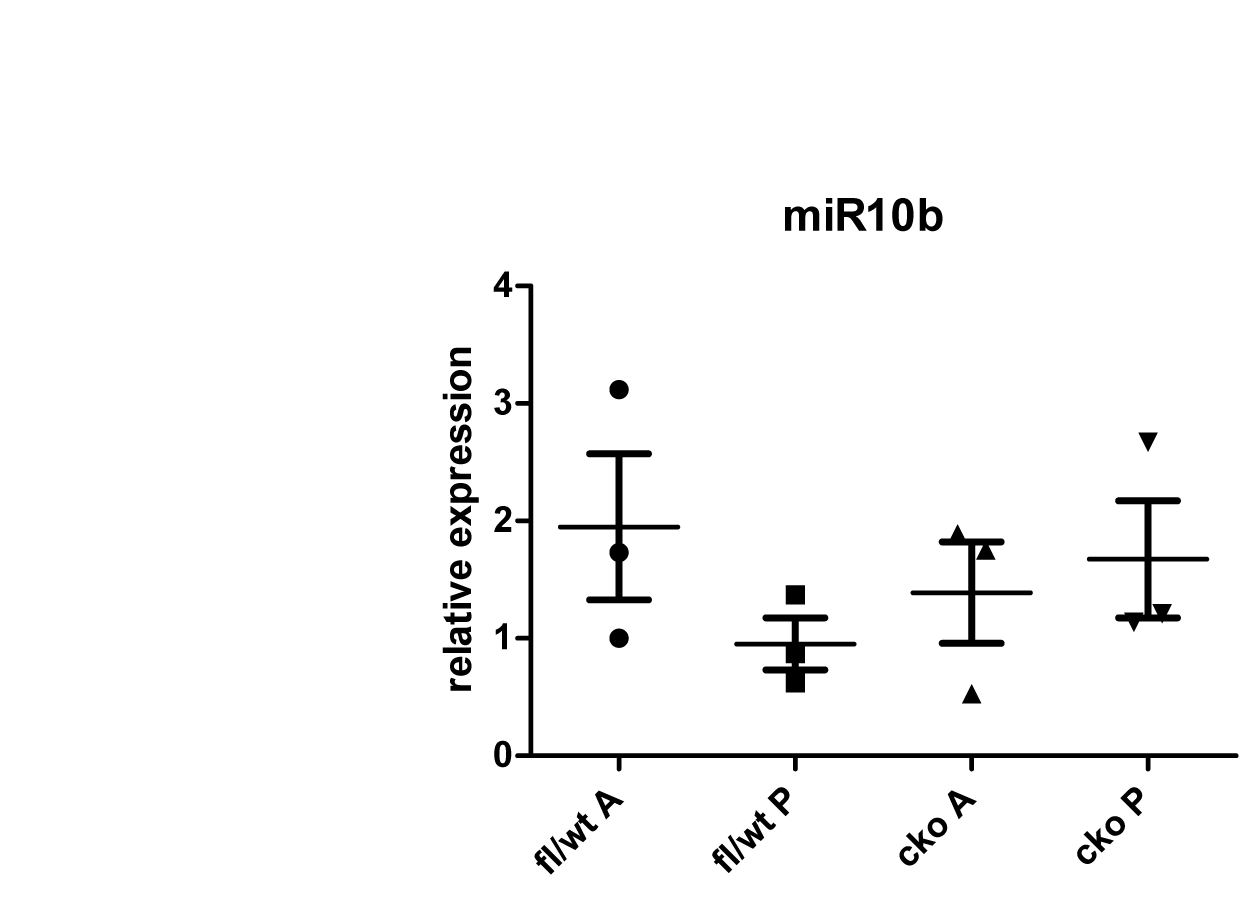

Supplement: Figure S2 — miR10b is not significantly downregulated in conditional mutant limb buds. qRT-PCR for miR10b in anterior (A) and posterior (P) halves of E11.5 forelimb buds dissected from control (fl/wt) and TAM E9.5 (cko) embryos. Reference gene was miR191. N = 3 for each genotype. (TIF) [file pone.0098945.s002.tif]
